# Antimicrobial Peptides in Biomedical Device Manufacturing

**DOI:** 10.3389/fchem.2017.00063

**Published:** 2017-08-24

**Authors:** Martijn Riool, Anna de Breij, Jan W. Drijfhout, Peter H. Nibbering, Sebastian A. J. Zaat

**Affiliations:** ^1^Department of Medical Microbiology, Academic Medical Center, Amsterdam Infection and Immunity Institute, University of Amsterdam Amsterdam, Netherlands; ^2^Department of Infectious Diseases, Leiden University Medical Center Leiden, Netherlands; ^3^Department of Immunohematology and Blood Transfusion, Leiden University Medical Center Leiden, Netherlands

**Keywords:** antimicrobial peptide, biomaterial-associated infection, biofilm, antimicrobial resistance, implant, device manufacturing

## Abstract

Over the past decades the use of medical devices, such as catheters, artificial heart valves, prosthetic joints, and other implants, has grown significantly. Despite continuous improvements in device design, surgical procedures, and wound care, biomaterial-associated infections (BAI) are still a major problem in modern medicine. Conventional antibiotic treatment often fails due to the low levels of antibiotic at the site of infection. The presence of biofilms on the biomaterial and/or the multidrug-resistant phenotype of the bacteria further impair the efficacy of antibiotic treatment. Removal of the biomaterial is then the last option to control the infection. Clearly, there is a pressing need for alternative strategies to prevent and treat BAI. Synthetic antimicrobial peptides (AMPs) are considered promising candidates as they are active against a broad spectrum of (antibiotic-resistant) planktonic bacteria and biofilms. Moreover, bacteria are less likely to develop resistance to these rapidly-acting peptides. In this review we highlight the four main strategies, three of which applying AMPs, in biomedical device manufacturing to prevent BAI. The first involves modification of the physicochemical characteristics of the surface of implants. Immobilization of AMPs on surfaces of medical devices with a variety of chemical techniques is essential in the second strategy. The main disadvantage of these two strategies relates to the limited antibacterial effect in the tissue surrounding the implant. This limitation is addressed by the third strategy that releases AMPs from a coating in a controlled fashion. Lastly, AMPs can be integrated in the design and manufacturing of additively manufactured/3D-printed implants, owing to the physicochemical characteristics of the implant material and the versatile manufacturing technologies compatible with antimicrobials incorporation. These novel technologies utilizing AMPs will contribute to development of novel and safe antimicrobial medical devices, reducing complications and associated costs of device infection.

## Biomaterial-associated infections

The use of medical devices, including catheters, artificial heart valves, prosthetic joints, and other implants, increased dramatically over the past century (Darouiche, 2004; Anderson and Patel, 2013; Kwakman and Zaat, 2013), and has become a major part of modern medicine and our daily life. With the aging society, the demand for medical devices to restore body functions and quality of life increases, and so do the numbers of cases of biomaterial-associated infection (BAI). The risk for BAI may in part be explained by the reduced efficacy of the local immune defense induced by the foreign body. In agreement, the number of bacteria required to cause an infection is significantly lower in the presence of a foreign body, such as a stitch or an implant, than when such devices are not present (Elek and Conen, 1957; James and Macleod, 1961; Noble, 1965; Taubler and Kapral, 1966; Zimmerli et al., 1982; Southwood et al., 1987). Another contributing factor is that the bacteria—often derived from the commensal skin flora or the hospital environment—can adhere to the foreign body, replicate, and form a biofilm from which they can invade the peri-implant tissues and cause an infection. The most common causative microorganisms in BAI are *Staphylococcus aureus* and *Staphylococcus epidermidis* (Anderson and Marchant, 2000; O'Gara and Humphreys, 2001; Zimmerli et al., 2004). Depending on the type of device and location of application, other coagulase-negative staphylococci, enterococci, streptococci, *Propionibacterium acnes*, and yeasts such as *Candida* spp., can also cause BAI (Waldvogel and Bisno, 2000; Holmberg et al., 2009). Infections following primary implant surgery occur in 0.5–1% of the patients receiving an artificial hip or knee and in over 5% of those receiving a prosthetic elbow or ankle implant (Zimmerli et al., 2004; Krenek et al., 2011). As treatment of BAI is complex, combinations of antibiotics, such as vancomycin or ciprofloxacin with rifampicin, are recommended. Such combinations show some efficacy against biofilms, although much higher concentrations of antibiotics are required than effective against planktonic cells (Saginur et al., 2006). Nevertheless, treatments with antibiotic combinations often fail with the only option being removal of the medical device (Burns, 2006). Catheters suspected for infection are removed and replaced by a new device at a different location, as re-implantation at the original site is strongly discouraged because of the high re-infection risk (Safdar et al., 2002). Revision surgery of infected orthopedic devices in most cases involves removal of the implant, thorough debridement of the infected site and prolonged (4–8 weeks) antibiotic treatment before a new implant is placed (Zimmerli, 2006). Still, revision surgery is associated with high frequencies of infection due to extensive surgical procedures and more severe tissue damage.

### Biofilm formation

Bacterial biofilm formation is considered to play a major role in the pathogenesis of BAI (Costerton et al., 1999; Holmberg et al., 2009; Anderson and Patel, 2013). Biofilm formation is initiated by bacterial cells attaching to the surfaces of medical devices. Subsequently, bacteria replicate and produce extracellular matrix forming complex communities consisting of bacteria, bacterial exopolysaccharides, proteins, extracellular DNA, and host proteins (Costerton et al., 1999). Bacteria in biofilms are considerably more tolerant to antibiotics and less accessible to cells and molecules of the human immune defense system than their planktonic counterparts (Otto, 2009; Chen et al., 2013). This might be due to the extracellular polymeric matrix of the biofilm, making the bacteria less accessible for phagocytes and effector molecules, and to the persister state of the bacteria. Persisters are metabolically-inactive, antibiotic tolerant bacteria that maintain the ability to multiply after antibiotic treatment (Harms et al., 2016), thus explaining the recurrence of BAI (Gerdes and Semsey, 2016; Fisher et al., 2017).

### Tissue colonization

Another important element in the pathogenesis of BAI is bacterial colonization of the tissue surrounding the implant (Boelens et al., 2000a; Ciampolini and Harding, 2000). *In vivo* studies showed that *S. epidermidis* applied on the surface of titanium implants, both as adherent cells and as a pregrown biofilm, rapidly relocated from the implants to the surrounding tissue (Riool et al., 2014). Similarly, large numbers of *S. aureus* were cultured from mouse tissues around infected titanium (Riool et al., 2017a,b) and silicon elastomer implants (de Breij et al., 2016). In a murine model of chronic osteomyelitis, *S. aureus* was found in osteoblasts and osteocytes, as well as in canaliculi of live cortical bone (de Mesy Bentley et al., 2017).

Bacterial invasion of the peri-implant tissue and subsequent development of infection is facilitated by dysregulation of the local immune response resulting from the presence of a foreign body. The phagocytic and intracellular killing activities of neutrophils and macrophages are reduced due to altered cytokine tissue levels in the presence of a biomaterial (Boelens et al., 2000a,b,c; Broekhuizen et al., 2010; Zimmerli and Sendi, 2011). In agreement, microscopical examination has revealed that many of the bacteria reside within these inflammatory phagocytes (Broekhuizen et al., 2010). Interestingly, studies in mice infected with *S. epidermidis* as well as in infected peri-catheter tissue biopsies obtained from deceased intensive care unit patients showed that bacteria present in tissue surrounding the implants had incorporated bromodesoxyuridine, demonstrating that the bacteria can replicate in the peri-implant tissue (Broekhuizen et al., 2010). Furthermore, bacteria may adapt to the tissue and intracellular micro-environment by the formation of so-called small colony variants. The presence of such intracellular small colony variants further complicates treatment as they are more resistant to antimicrobial compounds (Tuchscherr et al., 2010; Zaat, 2013).

### Antimicrobial resistance

In addition to the limited activity of antibiotics against biofilm-encased bacteria, persisters, and intracellular bacteria, the emergence of resistance among staphylococci as well as other bacterial species causing BAI constitutes a major challenge to the efficacy of (combinations of) conventional antibiotics. The emergence of multidrug-resistant (resistant to at least one agent in three or more antimicrobial classes), extensively drug-resistant (resistant to at least one agent in all but one or two antimicrobial classes), and pan-drug-resistant (resistant to all agents in all antimicrobial classes) pathogens, is accelerated by the selective pressure exerted by extensive use and abuse of antimicrobials (Magiorakos et al., 2012). Bacteria belonging to the so-called ESKAPE panel (*Enterococcus faecium, S. aureus, Klebsiella pneumoniae, Acinetobacter baumannii, Pseudomonas aeruginosa*, and *Enterobacter* species) are increasingly prevalent and resistant and thereby a particularly dangerous group of bacteria (Rice, 2008). Currently, the majority of hospital infections in the United States is caused by multidrug-resistant ESKAPE bacterial strains (Boucher et al., 2009). The World Health Organization recently endorsed a global action plan to tackle antibiotic resistance to avoid the dark scenario of a “post-antibiotic era” (Chan, 2015). One of the key objectives of this plan is to develop novel antimicrobial drugs with a mode of action different from those of current antibiotics.

## Antimicrobial peptides

Antimicrobial peptides (AMPs)—effector molecules of the innate defense of animals, plants, and microorganisms (Zasloff, 2002; Hancock and Sahl, 2006)—have recently attracted considerable interest as agents that may subvert many of the problems related to BAI, i.e., they display antimicrobial activity against bacteria resistant to antibiotics and residing within biofilms. A specialized biofilm-active AMPs database lists most of the published AMPs with anti-biofilm activity (Di Luca et al., 2015). AMPs are mostly amphipathic, cationic peptides that display antimicrobial activity against bacteria, fungi and (enveloped) viruses. They interact with specific constituents of the bacterial cell envelope resulting in depolarization, destabilization, and/or disruption of the bacterial plasma membrane leading to bacterial cell death within minutes (Pasupuleti et al., 2012). Due to the rapid and non-specific mechanisms of action, the risk of resistance development is generally thought to be low (Zasloff, 2002). Nonetheless, resistance to AMPs in bacteria does occur and several mechanisms of resistance have been described, including membrane and cell envelope structure alterations increasing positive charge, upregulation of efflux pumps, and proteolytic degradation of the peptides (Goytia et al., 2013; Ernst et al., 2015). For instance, resistance against the human cathelicidin LL-37 has been reported to involve degradation of the peptide by bacterial proteolytic enzymes, up-regulation of efflux pumps as well as bacterial-induced down-regulation of LL-37 expression in host cells (Bandurska et al., 2015). Under low calcium or magnesium ion concentrations, as in blood plasma, *P. aeruginosa* activates the *pmr* (polymyxin resistance) operon, which medicates the addition of N-arabinose to its lipopolysaccharide. This renders the outer surface of the bacterial cell more positively charged, repelling the cationic AMPs (Goytia et al., 2013). So, resistance of bacteria against AMPs is possible for several bacterial species, however development of such resistance against novel synthetic AMPs has not often been studied.

In addition to direct antimicrobial activity, AMPs display immunomodulatory activities. For example, they can prevent excessive activation of pro-inflammatory responses due to bacterial endotoxins such as lipopolysaccharide of Gram-negative bacteria, and peptidoglycan and lipoteichoic acid of Gram-positive bacteria. AMPs may improve clearance of bacterial biofilms by host defense systems (Mansour et al., 2014, 2015) as they may prevent derangement of immune responses after implantation of foreign bodies (Zaat et al., 2010; Heim et al., 2014, 2015). Other favorable characteristics of AMPs relate to wound healing (Nakatsuji and Gallo, 2012), angiogenesis (Salvado et al., 2013), and osteogenic activity (Kittaka et al., 2013; Zhang and Shively, 2013). Regarding the latter activity, it has been reported that in a trabecular bone growth *in vivo* study, cylindrical titanium implants coated with the antimicrobial peptide HHC36 had osteoconductive properties (Kazemzadeh-Narbat et al., 2012). Similarly, fusion peptide P15-CSP showed anti-biofilm activity and pro-osteogenic activity (Li et al., 2015) and LL-37 promoted bone regeneration in a rat calvarial bone defect model (Kittaka et al., 2013) and accelerated bone repair in NOD/SCID mice (Zhang and Shively, 2013).

Naturally occurring AMPs have been used as design templates for a large variety of synthetic AMPs, some of which have reached the stage of phase 2 and 3 clinical trials (Fox, 2013; Greber and Dawgul, 2016), such as OP-145 (Peek et al., 2009), LL-37 (Grönberg et al., 2014), Iseganan (IB-367; Mosca et al., 2000), Omiganan (MBI-226; Sader et al., 2004), and Pexiganan (MSI-78; Fuchs et al., 1998). With respect to the development of synthetic peptides for the treatment of BAI we will focus on a few of the most promising peptides. The synthetic peptide IDR-1018 prevented biofilm formation by *S. aureus* and various other species by blocking (p)ppGpp, which is a signal molecule in persister development (Harms et al., 2016) and biofilm formation (Mansour et al., 2015). In a murine model of *S. aureus* implant infection, IDR-1018 showed to be potentially useful in reducing orthopedic infections by recruiting macrophages to the infection site, blunting excess cytokine production and reducing osseointegration failures (Choe et al., 2015).

In an attempt to meet the requirements for the treatment of BAI as much as possible, a series of novel synthetic AMPs was recently developed based on two human AMPs, i.e., thrombocidin-1, the major antimicrobial protein of human blood platelets (Krijgsveld et al., 2000; Kwakman et al., 2011), and LL-37, a principal human AMP produced by mucosal epithelial cells and multiple immune cells. The LL-37-inspired peptide OP-145 (formerly designated as P60.4Ac; Nell et al., 2006) proved to be safe and efficacious for treatment of therapy-resistant otitis media patients (Peek et al., 2009). *In vitro*, OP-145 (de Breij et al., 2016), and the newer generation LL-37-inspired peptides SAAP-145 and SAAP-276 (Riool et al., 2017a) and the trombocidin-1-derived peptide TC19 (Zaat et al., 2014) inhibited biofilm formation by a clinical *S. aureus* BAI isolate in a dose-dependent fashion. The mode of action of these synthetic peptides may involve inhibition of adherence of bacteria to surfaces and/or reduction of expression of genes involved in biofilm formation, as has been reported for LL-37 (Overhage et al., 2008). These novel synthetic peptides all rapidly permeabilize the membrane of *S. aureus* bacteria (Riool et al., 2017a), explaining why they are highly effective against dividing as well as non-dividing, biofilm-encased bacteria whether or not resistant to antibiotics. Interestingly, these newer generation peptides display good bactericidal activity in the presence of human plasma, despite possible binding of the peptides to plasma components (de Breij et al., 2016). In contrast, the first generation AMP OP-145 showed strong reduction of antimicrobial activity in plasma *in vitro*. Despite this OP-145 proved to be effective in preventing *S. aureus* colonization of subcutaneous implants in mice and protected rabbits from experimental intramedullary nail-associated osteomyelitis (de Breij et al., 2016). Apparently, *in vitro* activities in the presence of human plasma do not necessarily predict the *in vivo* potency of AMPs.

The physical properties of synthetic AMPs, i.e., cationic charge and peptidic nature, present challenges to their biological stability and balance between antimicrobial efficacy and host cell toxicity. Fortunately, several solutions can be considered to address these issues. For example, PEGylation is a well-accepted method for minimizing cytotoxicity while maintaining antimicrobial activity of AMPs and reducing elimination of the peptides by the liver and kidneys (Morris et al., 2012). D-enantiomers—peptides that are comprised of unnatural amino acids—and (retro-)inverso peptides are insensitive to most peptidase activity (Guichard et al., 1994; Feng and Xu, 2016). In this connection, a series of modified HHC10 peptides were synthesized, including inverso-CysHHC10 (i.e., different stereo isomer). Inverso-CysHHC10 was stable in human serum, showed microbicidal activities at low micromolar concentrations against *Escherichia coli, S. aureus*, and *S. epidermidis* and was active in a polyethylene glycol (PEG)-based hydrogel in serum (Cleophas et al., 2014). Of note, serum may be a worst-case scenario for peptides, since Fibrinopeptide A peptides were degraded in serum, but not in fresh blood (Böttger et al., 2017). Serum may have a level of proteolytic activity not encountered in blood or plasma, since preparation of serum involves blood coagulation, which leads to activation of coagulation pathway proteases (Chambers and Laurent, 2002). Therefore, inhibition/degradation is best studied in plasma or fresh blood.

Another area of potential improvement of synthetic AMPs is that of intracellular antimicrobial activity, required to treat intracellular infections. In general, AMPs do not effectively penetrate host cells due to their high positive charge. Several cell-penetrating peptides have been developed for intracellular “delivery” of peptides. Such peptides may be utilized to deliver AMPs and PEGylated peptides into host cells to facilitate elimination of intracellular pathogens, e.g., staphylococci, residing within inflammatory and other cells, such as osteoblasts (Iwase et al., 2016).

## Preventive strategies

**Figure 1:**
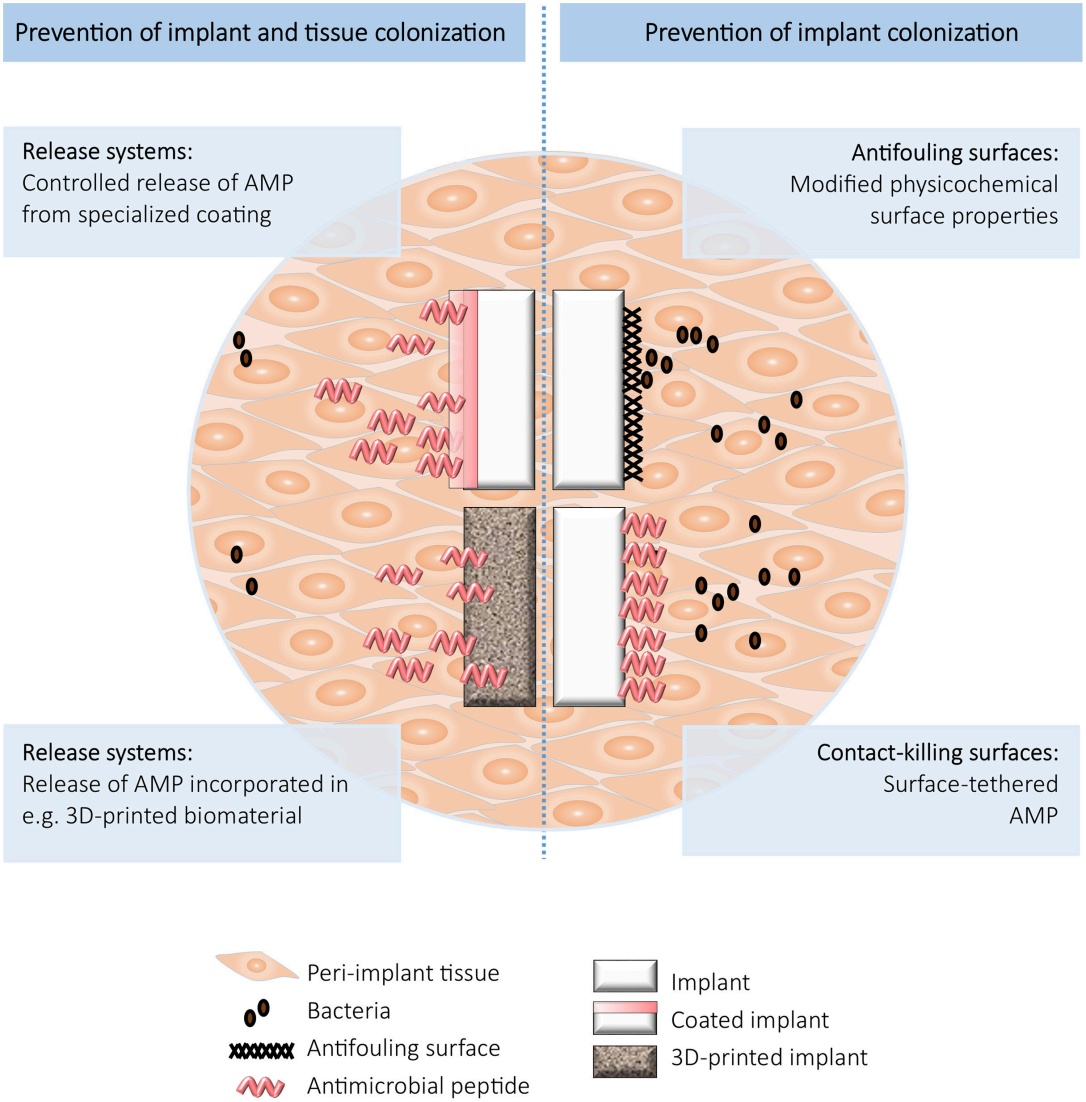
Schematic overview of the strategies to prevent implant (**Right**) and implant and tissue (**Left**) colonization.

For prevention of BAI, various types of antimicrobial biomaterials have been developed, including (i) antifouling surfaces, (ii) contact-killing surfaces, and (iii) surfaces which incorporate and release antimicrobials (Busscher et al., 2012). These approaches all have their benefits and limitations, which need to be taken into account when designing an antimicrobial strategy for a particular device (Brooks et al., 2013). Importantly, both biofilm formation on the implant and colonization of the peri-implant tissue need to be taken into consideration when designing preventive strategies against BAI. Here, we will discuss various combinations of these strategies and AMPs to prevent BAI (summarized in Figure 1).

### Antifouling surfaces

Already in 1987, Gristina suggested that adhering tissue cells and bacteria compete for a spot on the implant's surface, the so-called “race for the surface” concept (Gristina, 1987). In case this race is won by the bacteria, this will result in infection instead of tissue integration. Gristina also realized that colonization of the tissue around implants was another possible mechanism of infection (Gristina, 1987). Bacterial adhesion and subsequent biofilm formation may be prevented by modifying the physicochemical surface properties of biomaterials such as the surface charge, hydrophobicity/hydrophilicity, and surface chemistry. One strategy is to use hydrophilic polymer coatings, e.g., immobilized PEG, as applied on contact lenses, shunts, endotracheal tubes, and urinary catheters (Banerjee et al., 2011; Busscher et al., 2012). Another approach is functionalization of the surface with a dense layer of polymer chains commonly known as polymer brush coatings (Nejadnik et al., 2008; Neoh et al., 2013; Keum et al., 2017). Large exclusion volumes of tethered polymer chains result in surfaces difficult to approach by proteins or bacteria.

### Contact killing surfaces

Another approach to prevent implant colonization is the immobilization of AMPs on surfaces of medical devices, which can be performed with a variety of chemical techniques. An excellent overview of immobilization strategies has recently been published by Silva et al. (2016). There are several common “rules” for success. The structural characteristics important for the antimicrobial activity of the peptides should not be altered by the immobilization process. Length, flexibility, and kind of spacer connecting the peptide to the surface, orientation of the immobilized peptides, and the AMP surface density are additional important parameters (Costa et al., 2011). Interestingly, even short surface-attached peptides not likely to have a free interaction with the bacterial cytoplasmic membrane, have antimicrobial activity (Hilpert et al., 2009). This is thought to be due to destabilization of the bacterial membrane by displacement of positively charged counter-ions, disrupting the ionic balance, changing bacterial surface electrostatics, and activating autolytic enzymes (Hilpert et al., 2009). An example of a contact-killing surface is the hydrogel network with the covalently attached stabilized inverso-CysHHC10 peptide (Cleophas et al., 2014). This coating demonstrated high *in vitro* antimicrobial activity against *S. aureus, S. epidermidis*, and *E. coli*. Furthermore, brush coating molecules may also possess active functional groups with antimicrobial activity, e.g., by conjugation with the AMPs Tet20 (Gao et al., 2011a) and Tet213 (Gao et al., 2011b). Another example is polyurethane with a brush coating tethered with the AMP E6 for the prevention of catheter-associated infections (Yu et al., 2017). This surface coating reduced bacterial adhesion on the catheter surface in a mouse urinary catheter infection model. A variety of AMPs, like GZ3.27 (De Zoysa and Sarojini, 2017), GL13K (Chen et al., 2014; Zhou et al., 2015), SESB2V (Tan et al., 2014), bacitracin (Nie et al., 2016, 2017), hLF1-11 (Costa et al., 2014; Godoy-Gallardo et al., 2015), LL-37, Melimine, lactoferricin, and Mel-4 (Chen et al., 2016; Dutta et al., 2016) have been covalently coupled onto various surfaces, such as glass, silicon, and titanium, with different degrees of success (summarized in Table 1). Chimeric peptides comprised of both a titanium binding domain and an antimicrobial motif are also used to create contact-killing surfaces (Yucesoy et al., 2015; Liu et al., 2016; Yazici et al., 2016). Due to their titanium-binding domain, the peptides preferentially bind the implant, while the freely exposed antimicrobial domain is available for combatting invading bacteria. Titanium surfaces modified with these chimeric peptides were found to significantly reduce adhesion of different *Streptococcus* species, *S. aureus, S. epidermidis, P. aeruginosa*, and *E. coli*, compared to bare titanium. Immobilization of GL13K onto titanium dental implants even enabled osseointegration when tested in rabbit femurs (Chen et al., 2017). Another promising strategy is the development of multifunctional coatings by combining the well-known RGD cell adhesive sequence with the lactoferrin-derived AMP LF1-11, resulting in *in vitro* cell integration as well as inhibition of bacterial colonization by *S. aureus* and *Streptococcus sanguinis* (Hoyos-Nogués et al., 2017). Recently, others described a self-assembling coating of recombinant spider silk protein fused to the AMP Magainin I for different biomaterials, which reduced numbers of live bacteria on the coated surfaces (Nilebäck et al., 2017). It should be noted that not in all studies described above the absence of unbound peptide within the coating is verified. Thus, in those cases it cannot be excluded that the antimicrobial activity of the coating is caused by a combination of bound and released AMP.

**Table 1:** Overview AMP contact-killing surfaces.

**AMP**	**Coating type^1^**	**Surface^2^**	**Antimicrobial activity**	**References**
Bacitracin	Surface tethering	Titanium	Reduction surface adhesion by *S. aureus in vitro*, and reduction implant and tissue colonization by *S. aureus* in a rat femur implant infection model	Nie et al., 2016, 2017
Chimeric peptide^a^	Binding domain	Titanium	Surface bactericidal activity against *Streptococcus gordonii* and *S. sanguinis*^e^ *in vitro*	Liu et al., 2016
Chimeric peptide^b^	Binding domain	Titanium	Reduction surface adhesion by *Streptococcus mutans, S. epidermidis*, and *E. coli in vitro*	Yucesoy et al., 2015; Yazici et al., 2016
E6	Polymer brushes	PU	Reduction catheter surface colonization by *P. aeruginosa, S. aureus*, and *Staphylococcus saprophyticus in vitro* and by *P. aeruginosa* in mouse urinary catheter infection model	Yu et al., 2017
GL13K	Surface tethering	Titanium	Surface bactericidal activity against *S. gordonii* and *Porphyromonas gingivalis in vitro*	Chen et al., 2014; Zhou et al., 2015
GZ3.27^c^	Surface tethering	Titanium, glass, silicon	Surface bactericidal activity against *P. aeruginosa* and *E. coli in vitro*	De Zoysa and Sarojini, 2017
hLF1-11	Polymer brushes	Titanium	Surface bactericidal activity against *S. sanguinis* and *Lactobacillus salivarius in vitro*	Godoy-Gallardo et al., 2015
hLF1-11	Surface tethering	Titanium, chitosan	Reduction surface colonization by *S. aureus* (both surfaces) and *S. sanguinis* (chitosan) *in vitro*	Costa et al., 2014; Hoyos-Nogués et al., 2017
Inverso-CysHHC10	Hydrogel	PET	Surface bactericidal activity against *S. aureus, S. epidermidis*, and *E. coli in vitro*	Cleophas et al., 2014
Magainin I	Self-assembling silk	PS	Reduction surface adhesion by *S. aureus in vitro*	Nilebäck et al., 2017
Melimine	Surface tethering	Titanium	Reduction surface adhesion by *P. aeruginosa in vitro*, and reduction implant and tissue colonization by *S. aureus* in mouse and rat subcutaneous implant infection	Chen et al., 2016
Melimine, Mel-4, LFc^d^, LL-37	Surface tethering	pHEMA	Surface bactericidal activity against *P. aeruginosa* (LL-37, Mel-4, and Melimine) and *S. aureus* (Mel-4 and Melimine) *in vitro*	Dutta et al., 2016
SESB2V	Surface tethering	Titanium	Reduction tissue colonization by *S. aureus* and *P. aeruginosa* in a rabbit keratitis model	Tan et al., 2014
Tet20	Polymer brushes	Titanium	Surface bactericidal activity against *P. aeruginosa* and *S. aureus in vitro*, reduction surface adhesion by *S. aureus* in rat subcutaneous implant infection	Gao et al., 2011a
Tet213	Polymer brushes	Titanium	Surface bactericidal activity against *P. aeruginosa in vitro*	Gao et al., 2011b

1 *Surface tethering by covalent immobilization of AMP to surface; Chimeric peptide consists of titanium-binding domain and antimicrobial motif*.

2 *PET, polyethylene terephthalate; PU, polyurethane; pHEMA, poly-hydroxyethylmethacrylate; PS, polystyrene*.

a *Chimeric peptides consist of minTBP-1 and JPH8194 motifs*.

b *Chimeric peptides consist of TiBP(S)1–3 and E14LKK/H14LKK or KWKRWWWWR motifs*.

c *GZ3.27 with an added N-terminal cysteine is designated GZ3.163*.

d *LFc, lactoferricin*.

e *Formally known as Streptococcus sanguis, as mentioned in the reference*.

It should be noted that surface attachment of peptides does suffer from some disadvantages. The antimicrobial activity of the surface with immobilized AMPs is critically dependent on the chemical tethering procedure and the orientation of the covalently attached AMPs. The antimicrobial activity of the resulting coating may be strongly reduced compared to the activity of the peptide in free form (Bagheri et al., 2009; Onaizi and Leong, 2011; Dutta et al., 2016). Apart from this reduction of activity due to the tethering process, proteins, blood platelets, and dead bacteria may block the antimicrobial groups on the surface. Moreover, since the antimicrobial activity is restricted to the surface of the implant, there is a lack of antimicrobial impact on bacteria in the tissue surrounding the implant. Contact-killing surfaces will only eradicate bacteria that are in direct contact with the active surface, meaning that clearance of any bacteria further away from the surface will depend on efficient phagocytosis and systemic or local antibiotics. However, as mentioned before, due to the presence of a biomaterial the local host immune response is dysregulated, and therefore phagocytosed bacteria may not be killed and may even persist intracellularly (Boelens et al., 2000a,b).

### Release systems

As described above, the peri-implant tissue is an important niche for bacterial survival. Therefore, antimicrobial-releasing surfaces or coatings from which the antimicrobial agent also reaches this niche are preferred to prevent BAI. Antibiotic-releasing coatings are widely used for medical devices such as sutures and central venous catheters and urinary tract catheters. However, these coatings have two major disadvantages: (i) a patient may be infected with a bacterium resistant to the released antibiotic, and (ii) due to the local release a gradient of the antibiotic will be present near the implant thereby increasing the risk to select for resistant bacteria. Coatings releasing antibiotics for orthopedic devices remain mainly experimental (Lucke et al., 2003; Kälicke et al., 2006; Darouiche, 2007; Moojen et al., 2009; Alt et al., 2011). The first commercially available gentamicin-releasing intramedullary tibia nail has recently shown promising results in a first prospective study (Fuchs et al., 2011; Metsemakers et al., 2015; Alt, 2017). In view of the increasing development of antibiotic resistance among bacteria, the use of antibiotics in medical devices is discouraged by government regulatory agencies like the American Food and Drug Administration (FDA, 2007; Brooks et al., 2013). Obviously, coatings releasing antimicrobial agents that are less likely to induce resistance, such as AMPs, are preferred in view of both managing resistance development and compatibility with use of antibiotics for prophylaxis or treatment. To prevent the spread of bacteria from the implant surface to the surrounding tissue, and to eradicate bacteria contaminating tissue during surgery, a rapid initial release of antimicrobials is required. If this release is delayed, bacteria may “escape” into host cells before effective levels of the antimicrobial agent have been established. Subsequently, prolonged local release of the antimicrobial agent at sufficiently high concentrations will be required to eradicate any residual bacteria (Zilberman and Elsner, 2008; Emanuel et al., 2012).

Application of AMPs in antimicrobial surface coatings is a subject of increasing interest and different types of release-coatings have been described, including hydrogels, nanotubes, microporous calcium phosphate coatings, and polymer coatings (summarized in Table 2). Hydrogels with the AMP Cateslytin strongly adhere to dental implant surfaces. The hydrogels showed potent antimicrobial activities against *Porphyromonas gingivalis*, an important causative agent of peri-implantitis, without signs of toxicity (Mateescu et al., 2015). Another example is a gelatin-based hydrogel on titanium surfaces allowing for the controlled release of the short cationic AMP HHC36 preventing *S. aureus, S. epidermidis, E. coli*, and *P. aeruginosa* biofilm formation (Cheng et al., 2017).

**Table 2:** Overview AMP release coatings.

**AMP**	**Coating type^1^**	**Surface**	**Antimicrobial activity**	**References**
Cateslytin (CTL)	Hydrogel	Titanium, gingiva^b^	Surface bactericidal activity against *P. gingivalis in vitro*	Mateescu et al., 2015
GL13K	TiO_2_ nanotubes	Titanium	Antimicrobial activity against *F. nucleatum* and *P. gingivalis in vitro*	Li et al., 2017
HHC36	TiO_2_ nanotubes	Titanium	Bactericidal activity against *S. aureus* in solution and on surface *in vitro*	Ma et al., 2012
HHC36	Hydrogel	Titanium	Surface bactericidal activity against *S. aureus, S. epidermidis, E. coli*, and *P. aeruginosa in vitro*	Cheng et al., 2017
OP-145	PLEX	Titanium	Bactericidal activity against planktonic *S. aureus in vitro*, prevention of *S. aureus* BAI in a rabbit intramedullary implant infection model	de Breij et al., 2016
PSI 10	Microporous calcium phosphate	Magnesium alloy	Bactericidal activity against *S. aureus* in solution *in vitro*	Tian et al., 2015
SAAP-145, SAAP-276	PLEX	Titanium	Reduction implant and tissue colonization by *S. aureus* in a subcutaneous mouse implant infection model	Riool et al., 2017a
Tet213	Microporous calcium phosphate	Titanium	Bactericidal activity against *S. aureus* and *P. aeruginosa* in solution *in vitro*	Kazemzadeh-Narbat et al., 2010
Tet213	Collagen^a^	Titanium	Antimicrobial activity against *P. gingivalis* and *S. aureus* in solution *in vitro*	Shi et al., 2015

1 *PLEX, polymer-lipid encapsulation matrix; TiO_2_, titanium oxide*.

a *Biodegradable coating of Tet213 linked to collagen*.

b *Hydrogel adheres upon injection*.

Self-organized and vertically oriented titanium oxide nanotubes loaded with the broad spectrum AMP HHC36 showed *in vitro* bactericidal activity against *S. aureus* in liquid surrounding the nanotubular surface and reduced bacterial colonization on the surface ~200-fold (Ma et al., 2012). GL13K-eluting coatings on these titanium oxide nanotubes prevented growth of *Fusobacterium nucleatum* and *P. gingivalis* in an *in vitro* disk-diffusion assay (Li et al., 2017). *In vitro* release of Tet213 from microporous calcium phosphate coatings applied on titanium showed bactericidal activity against *S. aureus* and *P. aeruginosa* (Kazemzadeh-Narbat et al., 2010). In a similar approach, release of PSI 10 from microporous calcium phosphate coated magnesium alloy inhibited *S. aureus* growth *in vitro* and promoted *in vivo* bone repair (Tian et al., 2015). Furthermore, controlled release of Tet213 linked to collagen IV inhibited *S. aureus* biofilm formation *in vitro* (Shi et al., 2015). However, these types of coatings have not yet been tested *in vivo*.

Injection of the LL-37-inspired AMPs OP-145 (de Breij et al., 2016), SAAP-145, and SAAP-276 (Riool et al., 2017a) along subcutaneous implants in mice did not reduce the numbers of *S. aureus* in the surrounding tissue. This might be because the AMPs did not effectively penetrate the tissue or were not taken up by the host cells and thereby not capable of killing internalized bacteria. However, when these AMPs were released from Polymer-Lipid Encapsulation Matrix (PLEX) coatings, the numbers of viable *S. aureus* bacteria were reduced in the peri-implant soft tissue in mice (Riool et al., 2017a) and even in bone in a rabbit humerus intramedullary nail infection model (de Breij et al., 2016). This clearly illustrates the benefit of the PLEX coating technology allowing controlled and prolonged release of the AMPs at the implant-tissue-interface. The SAAP-276-PLEX-coated implants were able to significantly, but not completely, reduce the number of doxycycline-resistant *S. aureus* in the peri-implant tissue, in contrast to the doxycycline-PLEX coated implants which failed to reduce their numbers in the tissue (Riool et al., 2017a). This underlines the potency of SAAP-276-PLEX coatings in the fight against BAI caused by multidrug-resistant staphylococci.

Although the AMPs mentioned above reduced the colonization of the peri-implant tissue *in vivo* when released from a coating, they might still not be able to act against intracellular bacteria. Apparently, the rapid initial release of the AMPs killed the vast majority of the infecting bacteria, preventing biofilm formation on the implant surface as well as colonization of the tissue, thereby protecting both these sites against colonization. Treatment of infections featuring intracellular bacteria remains difficult, as observed with the conventional antibiotic vancomycin (Broekhuizen et al., 2008), and likely with the novel AMPs as well. A possible way to improve the intracellular entry of AMPs is by adding a specific domain (“tag”) to the peptides as a signal for uptake by the host cells (Splith and Neundorf, 2011; Ye et al., 2016). However, intracellular localization of bacteria does not seem to occur to a large extent when AMPs are used in BAI prevention, as shown for instance with the AMP-PLEX coatings described above. By directly killing the bacteria on the implant-tissue interface the AMPs prevented bacterial invasion into the tissue and internalization by and survival in host cells.

### Novel manufacturing techniques for biomaterials, a role for AMPs?

Several novel technologies are arising for manufacturing implants with particular focus on the possibility of personalization. We will briefly address additive manufacturing and electrospinning with regards to the strategies of incorporation of antimicrobial agents and potential for AMPs.

#### Additive manufacturing

Additive manufacturing (3D-printing) of medical devices is a major breakthrough that enables the production of implants customized in size and shape, and potentially with high porosity, thereby increasing the surface area. These aspects make this technique attractive for personalized implants. However, as with conventional implants, the 3D-printed implants are susceptible to infection. Therefore, different approaches are currently explored to develop 3D-printed medical devices with antimicrobial functionalities. For example, antimicrobials may be added by surface modification of the 3D-printed implants, using plasma electrolytic oxidation, also known as micro-arc oxidation (Fidan et al., 2017). In this process, a titanium oxide layer is generated and compounds or nanoparticles present in the electrolyte are incorporated in the growing surface oxide layer (Necula et al., 2009; Lara Rodriguez et al., 2014; Fidan et al., 2017). One antimicrobial agent often used for the implants is silver. Silver is used in numerous medical applications (Bach et al., 1999; Rupp et al., 2005; Osma et al., 2006; Kuehl et al., 2016) and has broad-spectrum antimicrobial activity (Bürgers et al., 2009; Sussman et al., 2015). In a recent study silver nanoparticles were embedded in the titanium oxide layer of 3D-printed titanium implants (van Hengel et al., 2017) using a plasma electrolytic oxidation protocol developed for conventional medical grade titanium implants (Necula et al., 2009, 2012). These 3D-printed implants released silver ions over time, and showed *in vitro* bactericidal activity against MRSA including prevention of biofilm formation, and eradicated MRSA in an *ex vivo* mouse femur implant infection model (van Hengel et al., 2017).

The antibiotics rifampin and vancomycin have been incorporated in 3D-printed calcium-phosphate scaffolds during manufacturing. Due to their local delivery these incorporated antibiotics rendered the scaffolds capable of controlling murine implant-associated bone infection (Inzana et al., 2015). A similar approach might very well be suitable to incorporate AMPs for local delivery, as an alternative for the use of conventional antibiotics. AMPs might also be incorporated in hydrogels to coat the 3D-printed implants, similar to approaches utilizing polymers to create antibiotic release systems (ter Boo et al., 2015, 2016).

Another novel approach currently explored for prevention or treatment of BAI is the use of gold nanoparticles with tethered AMPs to increase the *in vivo* stability of AMPs and decrease possible toxicity. This technology would be readily applicable to 3D-printed implants. Gold nanoparticles conjugated with the hydrophilic cationic peptide cecropin melittin (CM) demonstrated higher antimicrobial activity and stability in serum than the CM peptide in solution, The CM-gold nanoparticles had favorably low cytotoxicity for human cells and demonstrated high antimicrobial activity in mouse chronic wound infection and system infection models (Rai et al., 2016a,b).

#### Electrospinning

Electrospinning is an entirely different technique which offers many possibilities for manufacturing medical devices. An example is the electrospun prosthetic heart valve, which has reached the phase of advanced preclinical testing (Kluin et al., 2017). By electrospinning, biocompatible nanofibers can be produced that have a large surface area mimicking the extracellular matrix of the body. The porosity of the matrices may however allow colonization by bacteria. To reduce the risk of infection of electrospun materials, antimicrobial agents have been incorporated in the polymers used for the electrospinning process. Examples include antibiotics such as vancomycin and/or rifampicin (Waeiss et al., 2014; Song et al., 2016), moxifloxacin (Song et al., 2016), silver nanoparticles (Tian et al., 2013; Almajhdi et al., 2014), or combinations of silver nitrate and chlorhexidine (Song et al., 2016). Recently, studies have also reported on the use of AMPs to render electrospun materials antimicrobial. Poly(E-caprolactone) nanofibers have been loaded with the synthetic AMP inverso-crabrolin (Eriksen et al., 2013), and poly(vinyl alcohol) nanofibers with pleurocidin (Wang et al., 2015) or the antifungal peptide Cm-p1 (Viana et al., 2015). In view of the increasing importance of electrospinning applications in the medical field, this is an area where additional studies on the use of AMPs will be highly relevant and novel forms of AMP structures may be highly desired. In this respect a novel class of antimicrobial agents is emerging. This is the class of structurally nanoengineered antimicrobial peptide polymers (SNAPPs). In the form of 16- or 32-arm star-shaped peptide polymer nanoparticles, these SNAPPs showed *in vitro* activity at sub-micromolar concentrations against a wide panel of Gram-negative bacteria, including multidrug-resistant pathogens. They were effective *in vivo* against an multidrug-resistant strain of *A. baumannii* and did not induce resistance (Lam et al., 2016).

## Conclusions and future perspective

Prevention and treatment of BAI is a major medical challenge, in particular due to the involvement of biofilm-encased and intracellular multidrug-resistant bacteria. Synthetic AMPs, displaying broad spectrum activity including activity against multidrug-resistant pathogens, anti-biofilm activities, little/no development of resistance, and *in vivo* activity in preventing BAI, are important candidates. Tethering of these AMPs to the biomaterial surfaces, and particularly combining AMPs with formulations to release the peptides in a controlled fashion is expected to protect both the implant and the surrounding tissue, both for conventional implants and biomedical devices manufactured by 3D-printing and electrospinning.

## Author contributions

All authors listed have made a substantial, direct and intellectual contribution to the work, and approved it for publication.

### Conflict of interest statement

The authors declare that the research was conducted in the absence of any commercial or financial relationships that could be construed as a potential conflict of interest.

## Abbreviations

AMPs: antimicrobial peptides
BAI: biomaterial-associated infections
CM: cecropin melittin
PEG: polyethylene glycol
PET: polyethylene terephthalate
pHEMA: poly-hydroxyethylmethacrylate
PLEX: polymer-lipid encapsulation
matrixPS: polystyrene
PU: polyurethane
SAAP: synthetic antimicrobial and anti-biofilm peptide
TiO_2_: titanium oxide.
